# EPHA2, EPHA4, and EPHA7 Expression in Triple-Negative Breast Cancer

**DOI:** 10.3390/diagnostics12020366

**Published:** 2022-02-01

**Authors:** Ilias Nikas, Constantinos Giaginis, Kalliopi Petrouska, Paraskevi Alexandrou, Artemis Michail, Panagiotis Sarantis, Gerasimos Tsourouflis, Eugene Danas, Alexandros Pergaris, Panagiotis K. Politis, Lydia Nakopoulou, Stamatios Theocharis

**Affiliations:** 1 First Department of Pathology, Medical School, National and Kapodistrian University of Athens, 115 27 Athens, Greece; I.nikas@euc.accy (I.N.); ppetrouska@hotmail.com (K.P.); parialexandro@yahoo.com (P.A.); psarantis@med.uoa.gr (P.S.); eugenedanas@gmail.com (E.D.); alexperg@yahoo.com (A.P.); lnako@med.uoa.gr (L.N.); 2 School of Medicine, European University Cyprus, Nicosia 2404, Cyprus; 3 Department of Food Science and Nutrition, School of Environment, University of Aegean, Myrina, 811 00 Lemnos, Greece; cgiaginis@aegean.gr; 4 Center for Basic Research, Biomedical Research Foundation of the Academy of Athens, 4 Soranou Efesiou Str., 115 27 Athens, Greece; artemisbio@gmail.com (A.M.); ppolitis@bioacademy.gr (P.K.P.); 5 Second Department of Propedeutic Surgery, Laikon Hospital, Medical School, National and Kapodistrian University of Athens, 115 27 Athens, Greece; gtsourouflis@med.uoa.gr

**Keywords:** biomarker, basal-like breast cancer, EPH, ephrin, targeted therapy

## Abstract

Ongoing research continues to elucidate the complex role of ephrin receptors (EPHs) and their ligands (ephrins) in breast cancer pathogenesis, with their varying expression patterns implied to have an important impact on patients’ outcome. The current study aims to investigate the clinical significance of EPHA2, EPHA4, and EPHA7 expression in triple-negative breast cancer (TNBC) cases. EPHA2, EPHA4, and EPHA7 protein expression was assessed immunohistochemically on formalin-fixed and paraffin-embedded (FFPE) TNBC tissue sections from 52 TNBC patients and correlated with key clinicopathologic parameters and patients’ survival data (overall survival (OS); disease-free survival (DFS)). EPHA2, EPHA4, and EPHA7 expression was further examined in TNBC cell lines. EPHA2 overexpression was observed in 26 (50%) of the TNBC cases, who exhibited a shorter OS and DFS than their low-expression counterparts, with EPHA2 representing an independent prognostic factor for OS and DFS (*p* = 0.0041 and *p* = 0.0232, respectively). EPHA4 overexpression was associated with lymph node metastasis in TNBC patients (*p* = 0.0546). Alterations in EPHA2, EPHA4, and EPHA7 expression levels were also noted in the examined TNBC cell lines. Our study stresses that EPHA2 expression constitutes a potential prognostic factor for TNBC patients. Given the limited treatment options and poorer outcome that accompany the TNBC subtype, EPHA2 could also pose as a target for novel, more personalized, and effective therapeutic approaches for those patients.

## 1. Introduction

Breast cancer makes up the leading cause of cancer incidence and mortality in women worldwide [1]. It does not represent a single but an heterogeneous group of malignancies with varying molecular signatures, morphology, and clinical behavior [2]. Data from gene expression studies have clustered breast cancers into four main intrinsic subtypes—luminal A, luminal B, HER2-enriched, and basal-like breast cancers (BLBCs)—that display differences in prognosis and response to therapy [3,4,5,6,7,8,9]. Triple-negative breast cancers (TNBCs), characterized by a combined estrogen receptors (ER), progesterone receptors (PR), and human epidermal growth factor receptor 2 (HER2)-negative immunophenotype, make up 12–17% of the entire malignant breast population and show an epidemiological connection to younger women, African American race, and breast cancer gene 1 (*BRCA1*) mutations; most fall into the BLBC subtype [10,11,12]. TNBCs similarly appear heterogeneous at the molecular, morphologic, and clinical levels [13,14]. They behave aggressively, showing dismal prognosis with frequent recurrences and shorter survival rates compared to the luminal and HER2-positive breast cancers [10,12]. Notably, their negative immunophenotype means that targeted medication, such as tamoxifen and trastuzumab, provides no benefit to these patients; therefore, traditional cytotoxic chemotherapy is the only option which, although effective initially, fails to maintain a long-term response. TNBCs’ bad prognosis, combined with the lack of any specific targeted therapies, has resulted in extensive ongoing research that attempts to discover prognostic and/or predictive biomarkers against TNBCs or, at least, any subgroup of them [10,12,13,14].

Ephrin receptors (EPHs) have shown great promise as potential biomarkers against various cancer types. This family of receptor tyrosine kinases (RTKs) comprises 14 cell-bound members divided into two classes, A and B, based on their sequence similarity and binding affinity to their ligands, ephrins; in a similar manner, ephrins are also cell-bound proteins and include ephrin-A and ephrin-B classes. Nine class A EPHs (EPHA1–EPHA8, EPHA10) and five class B EPHs (EPHB1–EPHB4, EPHB6) preferentially bind five ephrin-A and three ephrin-B classes, respectively [15,16]. The EPH/ephrin system is involved into key regulatory processes of human physiology; it highlights axon guidance and synaptic plasticity in the nervous system and is implicated in angiogenesis, vascular remodeling, and homeostasis of various tissues. In addition, this cell–cell communication system is implicated in several pathologic processes including neurodegenerative disorders, viral infections, and cancer. Indeed, its deregulation affects tumor growth, disease progression, metastasis, and neovascularization by disrupting critical signaling transduction pathways. EPHs’ and ephrins’ aberrant expression characterize not only the tumor cells but also the tumor microenvironment, where endothelial cells have mostly been investigated; this makes the EPH/ephrin system an appealing candidate for targeted intervention [15,16,17,18,19,20].

Various research groups have reported EPHs’ upregulation in the pathogenesis of a plethora of malignant neoplasms including lung, prostate, colon, pancreatic, esophageal, ovarian, thyroid, tongue and hepatocellular carcinomas in addition to gliomas, melanomas, neuroblastomas and leukemias [15,19,21,22]. Their expression in breast cancer has been investigated using cell lines, animal models, and human tissue samples, and correlated with patients’ prognosis [19,23,24,25,26,27,28,29,30] EPHA2, EPHB4, and EPHB6 are the receptors most extensively studied so far [15,17,18,20,28,31]. However, besides the abundance of studies on breast cancer models, respective EPHA expression studies on TNBC human material have only lately been described [32,33]. Our study aimed to investigate the expression pattern of EPHA2, EPHA4, and EPHA7 by immunohistochemistry (IHC) on formalin-fixed and paraffin-embedded tissue samples (FFPE) from TNBC patients and to correlate it with tumors’ clinicopathological characteristics, proliferative capacity (Ki-67 labeling index (LI)) and patients’ OS and DFS. Additionally, the expression of EPHA2, EPHA4, and EPHA7 was examined in established TNBC cell lines.

## 2. Materials and Methods

### 2.1. Patient Selection

This investigation was conducted in accordance with the principles of the Declaration of Helsinki and an approval from the Institutional Review Board of the University of Athens Medical School was obtained.

Overall, 52 archived invasive breast carcinoma histologic surgical samples from an equal number of female patients, aged from 32 to 84 years (mean: 57 years) were included in our study. None had received any neoadjuvant radiation or chemotherapy. All patients were treated with the same therapeutic strategy and received no targeted molecular therapies. Associated clinicopathologic characteristics including age, menopausal status, histologic type, grade, nuclear grade, tumor size, lymph node status, stage, Ki-67 LI, and patient survival data (OS; DFS) were also retrieved [34]. 

Samples were processed routinely. Breast cancer tissue samples were first fixed in 10% neutral buffered formalin, embedded in paraffin, and then cut in a microtome to generate tissue slides. The latter were subsequently stained with hematoxylin and eosin (H&E), examined by light microscopy and classified as ductal or lobular based on standard WHO histologic criteria (International Agency for Research on Cancer and World Health Organization 2012); thirty-four of them were typed as ductal and 18 as lobular. All tumor samples were graded according to the “Nottingham Histologic Score System” into three grades (I, II, and III) considering the amount of tubule formation, nuclear grade, and mitotic activity [35,36]. They were also staged in agreement with the American Joint Committee on Cancer (AJCC) TNM system based on tumor size, lymph node status, and the presence or absence of distant metastases [37]. Each breast cancer sample of this cohort was categorized as either stage I, II, or III, whereas no case was categorized as stage IV (none presented with a distant metastasis). All 52 samples were classified as TNBCs following the recommended criteria proposed at the latest St. Gallen International Breast Cancer Conferences [8,9]. 

### 2.2. Immunohistochemistry

IHC was performed on 4 μm FFPE breast tissue sections with commercially available rabbit polyclonal primary IgG antibodies against EPHA2 (H-120; sc-924; dilution 1:100), EPHA4 (H-77; sc-D-4; dilution 1:100), and EPHA7 (C-19; sc-1015; dilution 1:100) (Santa Cruz Biochemicals, Santa Cruz, CA, USA). Tissue sections were dewaxed in xylene and were brought to water through graded alcohols. Antigen retrieval (citrate buffer at pH 6.1 and microwave heating) was then performed. To remove the endogenous peroxidase activity, sections were treated with freshly prepared 0.3% hydrogen peroxide in methanol in the dark for 30 min (min) at room temperature. Nonspecific antibody binding was blocked using a specific blocking reagent for rabbit primary antibodies (Sniper, Biocare Medical, Walnut, Creek, CA, USA) for 5 min. The sections were then incubated for 1 h (h), at room temperature, with primary antibodies, diluted 1:100 in phosphate buffered saline (PBS). After washing three times with PBS, the sections were incubated at room temperature with biotinylated linking reagent (Biocare Medical) for 10 min, followed by incubation with peroxidase-conjugated streptavidin label (Biocare Medical) for 10 min. The resultant immune peroxidase activity was developed in 0.5% 3,3′-diaminobenzidine hydrochloride (DAB; Sigma, Saint Louis, MO, USA) in PBS containing 0.03% hydrogen peroxide for 5 min. Sections were then counterstained with Harris’s hematoxylin and mounted in Entellan (Merck, Darmstadt, Germany). Negative controls were generated by omitting the primary antibody and substituting it with irrelevant antiserum. Pancreatic, thyroid, and lung malignant tissue samples with already known EPHs’ overexpression from our previous experiments were used as positive controls [38,39,40]. 

Status of the proliferative marker Ki-67 was assessed with immunohistochemistry using a mouse anti-human Ki-67 antigen (IgG1k antibody, clone MIB-1; Dakopatts, Glostrup, Denmark) and classified into two categories (above and below median value), based on the percentage of the positively stained tumor nuclei [38,39,40,41].

IHC evaluation of EPHA2, EPHA4, and EPHA7 was performed by two independent surgical pathologists (S.T. and P.A.) in a blinded fashion to assess expression at the protein level. At least 1000 epithelial tumor cells were counted for each case and a 5% cut-off of positively stained malignant cells was needed to categorize each sample as “positive”. The scoring system for each immunostain was set based on the percentage of the stained tumor cells (0: 0–4% of tumor cells positive; 1: 5–24% of tumor cells positive; 2: 25–49% of tumor cells positive; 3: 50–100% of tumor cells positive) and the intensity of the immunostain (0: negative staining; 1: mild staining; 2: intermediate staining; 3: intense staining). A total score ≥ 3 was regarded as high (overexpression), whereas a total score of 0–2 was regarded as low expression.

### 2.3. Cell Culture

The human breast cancer cell lines (MDA-MB-231, MDA-MB-468, and MDA-MB-453) were cultured in the recommended medium (by ATCC) supplemented with 10% FBS (Biosera), 1% streptomycin/penicillin (Invitrogen, Waltham, MA, USA), and incubated in a 37 °C humidified incubator with 5% CO_2_. The human mammary gland cell line (MCF10A) was also cultured in the recommended medium (by ATCC) supplemented with 5% horse serum (Gibco, Waltham, MA, USA), 1% streptomycin/penicillin (Invitrogen), 10% insulin, 1% epidermal growth factor, 100 ng/mL cholera toxin, and incubated in a 37 °C humidified incubator with 5% CO_2_.

MDA-MB-231 is triple-negative B (TNB), MDA-MB-468 is triple-negative A (TNA), and MDA-MB-453 is ER and PR negative and Her2 positive. MCF10A are non-malignant breast epithelial cells that we used as controls for our study.

### 2.4. Western Blot Analysis

Total protein was isolated from cultured MDA MB 231, MDA MB 468, and MDAMB453 TNBC cells as well as from cultured MCF10A fibrocystic disease cells with the lysis buffer RIPA plus protease inhibitor cocktail. The homogenates were centrifuged at 17,000× *g* for 15 min at 4 °C. The supernatants were collected, and protein concentration was measured with the Bradford protein assay (Bio-Rad protein assay, Hercules, CA, USA). Thirty micrograms of protein samples were loaded each time into SDS-PAGE gels and transferred to nitrocellulose membranes (Whatman, Little Chalfont, UK) using the semi-dry transfer system (Bio-Rad). The membranes were blocked with 5% dry milk dissolved in Tris-buffered saline (1×) containing 0.1% Tween-20 for 1 h at room temperature (RT). The membranes were incubated with primary antibodies at 4 °C overnight followed by secondary antibodies for 1.30 h at RT. The primary antibodies in the Western blot were rabbit anti-EPHA2 (Santa Cruz, CA, USA, sc-924), mouse anti-EPHA4 (SantaCruz, sc-D-4), rabbit anti-EPHA7 (Santa Cruz, sc-1015), and mouse anti-beta actin (Sigma, A5441). The secondary antibodies were rabbit anti-mouse IgG (Sigma, A9044) (1:20,000 dilution) and goat anti-rabbit IgG (Sigma, A6154) (1:10,000 dilution).

## 3. Statistical Analysis

The Fisher’s exact test was used to correlate the EPHA2, EPHA4, and EPH7 protein expression by immunohistochemistry with the clinicopathologic parameters listed in Table 1, Table 2 and Table 3. The log-rank test was used to compare the differences between the survival curves constructed with the Kaplan–Meier method at univariate level. A Cox proportional-hazard regression model was developed to evaluate the association between the potential prognostic markers, OS and DFS, at a multivariate level. A *p*-value less than 0.05 was considered the limit of statistical significance. A *t*-test was performed for Western blot analysis, and SPSS for Windows software was used for all analyses (SPSS Inc., 2003, Chicago, IL, USA).

## 4. Results

**Figure 1:**
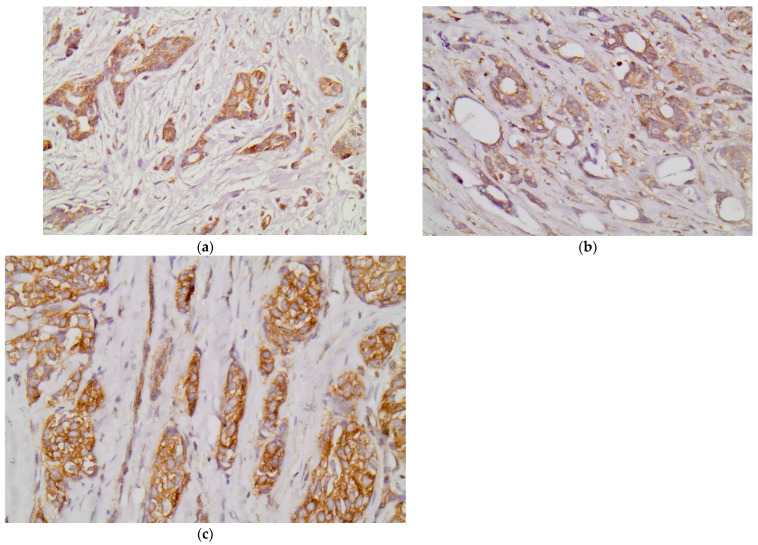
Representative positive immunostainings for EPHA2 (**a**), EPHA4 (**b**), and EPHA7 (**c**) in selected triple-negative breast cancer samples (200×).

High expression (overexpression) of EPHA2, EPHA4, and EPHA7 was noted in 26 (50%), 25 (48.1%), and 32 (61.5%) out of the 52 examined cases, respectively. Figure 1a–c shows representative positive immunostainings for EPHA2, EPHA4, and EPHA7 in selected TNBC tissue samples.

In the cross-tables, high EPHA2 expression was positively correlated with tumor proliferative capacity (Ki-67 LI) (Table 1; *p* = 0.0115), whereas no other significant associations were noted (Table 1). High EPHA4 expression was observed more frequently in TNBC cases accompanied by lymph node metastasis (Table 2; *p* = 0.0546), although insignificantly (Table 2). High EPHA7 expression was not significantly correlated with any of the examined clinicopathological parameters although a trend for nuclear grade was noted (Table 3; *p* = 0.0889). 

**Figure 2:**
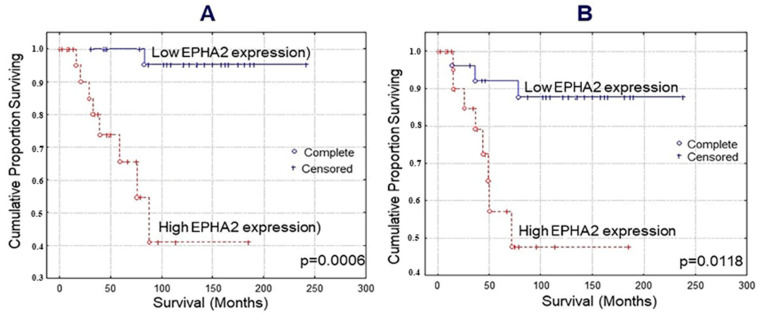
Kaplan–Meier survival analysis for EPHA2 high and low expression in 52 triple-negative breast cancer (TNBC) patients: overall survival (**A**) and disease-free survival (**B**).

Kaplan–Meier survival curves indicated that TNBC patients with EPHA2 overexpression exhibited shorter OS and DFS than those with low expression (Figure 2; log-rank test; *p* = 0.0006 and *p* = 0.0118, respectively). Multivariate analysis identified EPHA2 expression as independent prognostic factor for OS and DFS (Table 4 and Table 5; Cox-regression analysis; *p* = 0.0041 and *p* = 0.0232, respectively). Likewise, multivariate analysis identified Ki-67 status as an independent prognostic factor for disease-free but not for overall survival (Table 4 and Table 5 Cox-regression analysis; *p* = 0.0481 and *p* = 0.0808, respectively). EPHA4 and EPHA7 expression did not reach statistical significance regarding OS and DFS (data not shown). 

**Figure 3:**
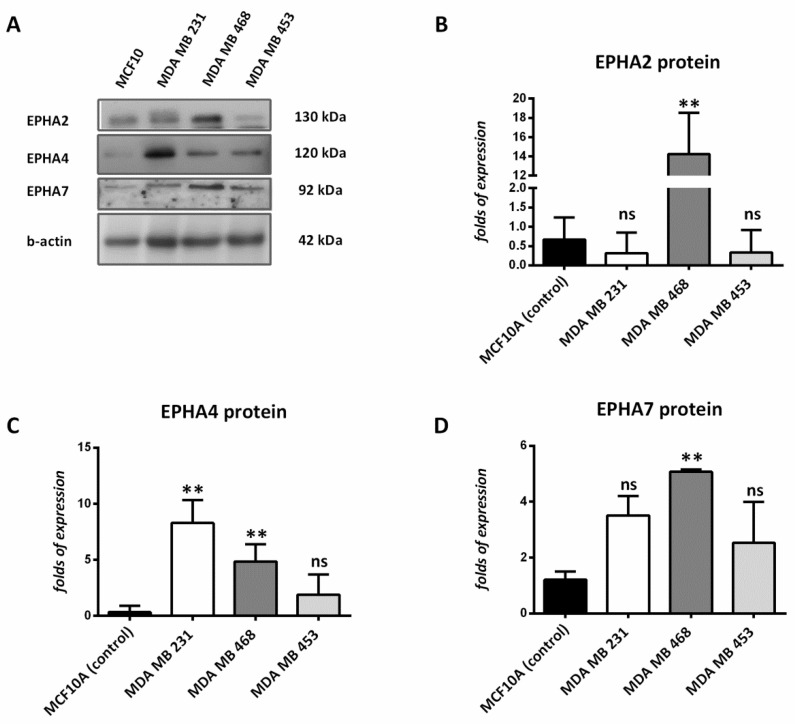
Protein expression analysis of EPHA2, EPHA4, and EPHA7 in MCF10A, MDA MB 231, MDA MB 468, and MDA MB 453 cell lines. (**A**) Western blot analysis of EPHA2, EPHA4, and EPHA7 protein expression in MCF10A, MDA MB 231, MDA MB 468, and MDA MB 453 cell lines. (**B**) Quantification of EPHA2 blots for MCF10A, MDA MB 231, MDA MB 468, and MDA MB 453 cell lines. Beta-actin was used as protein loading control. Data represent the mean and standard deviation from three independent experiments. (**C**) Quantification of EPHA4 blots for MCF10A, MDA MB 231, MDA MB 468, and MDA MB 453 cell lines. Beta-actin was used as protein loading control. Data represent the mean and standard deviation from three independent experiments. (**D**) Quantification of EPHA7 blots for MCF10A, MDA MB 231, MDA MB 468, and MDA MB 453 cell lines. Beta-actin was used as protein loading control. Data represent the mean and standard deviation from three independent experiments *p* < 0.05 and ** *p* < 0.01; ns indicates non-significance.

Furthermore, Western blot analysis for EPHA2, EPHA4, and EPHA7 was performed in three different human breast cancer cell lines. EPHA2, EPHA4, and EPHA7 protein expression was notably upregulated in the TNBC cell lines MDA MB 231 and MDA MB 468 compared to the normal one, MCF10A. On the other hand, no significant alterations were detected in the HER2 (+) breast cancer cell line, MDA MB 453. EPHA2 protein expression was found to be increased in the basal-type TNBC cell line MDA MB 468. The basal-like subtype of TNBC presents a significantly higher proliferation rate and is considered to be one of the most aggressive breast cancer subtypes. EPHA4 protein was significantly overexpressed in both TNBC cell lines, MDA MB 231 and MDA MB 468, when compared to the normal one (MCF10A). EPHA7 overexpression was found in the basal-like TNBC cell line MDA MB 468 (Figure 3).

## 5. Discussion

Besides their role in embryogenesis and tissue homeostasis, various EPH members are implicated in the processes of oncogenesis, tumor growth, progression, metastasis, and angiogenesis [15,18,19,20,22]. Investigation of the role of the EPH/ephrin system in breast cancer has mainly focused on the receptors EPHA2, EPHB4, and EPHB6 [15,17,18,28,42,43]. Notably, there are only a few EPHA expression studies on TNBC clinical tissue samples [32,33], although TNBC is accompanied by poor prognosis and lack of any targeted therapy. Establishing relevant clinical significance (correlation of various EPHs’ expression on human material with clinicopathologic characteristics and patient survival) is a fundamental step prior to the development of personalized therapies for TNBCs; it is also crucial for identifying potential subgroups of patients that might profit from such treatments [19,26].

In this study, we demonstrated a high EPHA2 protein expression in half of our TNBC breast cancer tissue samples. Furthermore, an inverse correlation, at a significant level, of high EPHA2 expression with OS and DFS in TNBCs was noted, establishing EPHA2 overexpression as a strong and independent dismal prognostic factor at both univariate and multivariate levels. We also link EPHA2 overexpression with tumor proliferative capacity (Ki-67 LI). Therefore, EPHA2 could be considered as a potential prognostic biomarker and therapeutic target towards TNBC patients. Secondarily, we linked, at an insignificant level, high EPHA4 expression with lymph node invasion and reported a trend between high EPHA7 expression and nuclear grade, albeit we showed no significant associations concerning each of these two EPHs and OS or DFS.

Two studies have focused on EPHA members’ expression specifically on patient-derived basal-like/TNBC material and associated it with clinicopathological parameters and survival. Similar to our group, Song et al. correlated EPHA2 overexpression with poor survival in basal-like/TNBC cases; they also found that EPHA2 was overexpressed in TNBC compared to benign breast cases by using IHC on tissue microarrays [32]. In addition, Hachim et al. linked overexpressed EPHA4 with higher tumor grade, stage, the basal-like breast cancer intrinsic subtype, and dismal prognosis [33].

Concerning breast cancer human material in general, some recent studies have attempted to link the expression of EPHA2, EPHA4, and EPHA7 with clinicopathologic parameters and/or survival. Martin et al. and Zhuang et al. used data mining and identified EPHA2 overexpression at the mRNA level as an adverse prognostic factor in ER- and HER2-positive breast cancer patients, respectively [23,44]. Brantley-Sieders et al. found EPHA2, EPHA4, and EPHA7 upregulated at both mRNA and protein levels; they also correlated EPHA2 overexpression with poor OS and DFS. Notably, they highlighted that measuring the accompanying ephrins’ expression might have clinical significance by reporting the loss of ephrin-A1 expression in EPHA2-positive breast cancer tissues accompanied by lymph node metastasis [26]. Edwards et al. linked EPHA2 overexpression with metastatic disease and dismal prognosis [45] and Youngblood et al. the overexpression of its phosphorylated form (pS897) with shorter survival in patients that present with lymph node metastases [46]. Last, Husa et al. quantified the mRNAs of 21 EPH/ephrin members on 65 breast carcinoma tissue samples with lymph node infiltration and used unsupervised hierarchical cluster analysis to group these cases into two clusters. EPHA2 and EPHA4, among others, were associated at a significant level with the cluster groups, while the cluster with the EPHs/ephrins overexpression was significantly linked to a worse prognosis. EPHB2 appeared as a strong and independent prognostic factor of poor outcome in the multivariate analysis [29]. 

Investigations from various development groups have provided compelling evidence about the role of EPHA2 in breast cancer. Despite its low expression in normal mammary epithelium, EPHA2 is overexpressed in the majority of breast cancers [19,28]. Ogawa et al. used both xenograft models and human tissue samples to demonstrate its upregulation, together with its ligand ephrin-A1, in the vasculature of mammary tumors, proposing EPHA2 as a possible anti-angiogenic therapeutic target [24]. Zelinski et al. displayed that EPHA2 is overexpressed at the protein level in breast tumor clinical samples, also cell lines, compared to benign epithelium and is able by itself to induce oncogenesis in MCF-10A cells [23]. Fox and Kandpal et al. correlated EPHA2 overexpression with breast oncogenesis and tumor progression in selected cell lines [47]. Another team reported that EPHA2 overexpression promotes breast cancer progression in synergy with HER2, suggesting that its therapeutic targeting could be of clinical value in HER2-positive mammary carcinomas. They also showed that its targeted disruption hinders oncogenesis and lung metastasis in the MMTV-Neu transgenic model [27]. Apart from breast cancer, EPHA2 overexpression has also been associated with poor prognosis in several other types of malignant tumors including ovarian, cervical, colorectal, vulvar, gastric, lung, oral, endometrial, esophageal, renal cell carcinomas, and gliomas [48,49,50,51,52,53,54,55,56,57,58]. 

Compared to EPHA2, research data concerning other EPHA members are far more limited. Overexpression of EPHA4 and EPHA7, as discussed earlier, has been associated with worse prognosis in breast cancer [26,33] as well as glioma [59]. Overexpression of EPHA4 is associated with poor prognosis in patients with gastric cancer [60]. High expression of EPHA4 but reduced expression of EPHB2 is linked with liver metastasis in human colon carcinomas [50], while high EPHA7 protein expression is associated with worse prognosis in patients with pancreatic cancer [39]. In contrast, overexpression of EPHA4 and EPHA7 is correlated with improved outcome in lung cancer [38,61]. High expression of EPHA7 at the protein level is linked with increased OS and DFS in patients with mobile tongue carcinoma [41].

Our present study indicates that among the EPHs examined (EPHA2, EPHA4, and EPHA7), EPHA2 constitutes a crucial prognostic factor for TNBC patients. In vitro experimental results are in agreement with the immunohistochemical data regarding EPHA2 expression in patients’ tissue samples and the Kaplan–Meier analysis, implying high EPHA2 expression as a poor prognostic factor.

However, the EPH/ephrin system does not function conventionally, and this represents the main problem towards designing effective drugs. It is capable of bidirectional signaling, forward and reverse, in a ligand-dependent manner through cell–cell contact (this means both EPHs and ephrins can initiate intracellular signaling) besides cross-talk with other signal transduction pathways in a ligand-independent manner [15,16,62]. Interestingly, forward signaling seems to suppress tumorigenesis; for instance, ephrin-A1 binds EPHA2 and inhibits a number of oncogenic signaling pathways [15,16,63]. Forward signaling also promotes angiogenesis [15,16]. In contrast, EPHs promote malignant transformation and progression mainly via a ligand-independent manner through their interplay with other signaling pathways. High unengaged EPHA2 levels interact with key pathways (Ras/Raf/Mek/Erk; PI3K/Akt/mTOR) connected to proliferation, growth, and survival normally inhibited with forward signaling [15,28,62]. A main reason for this might be the loss of the e-cadherin molecule, which causes cellular dissociation and prevents EPH/ephrin-suppressing interaction [15,28,51,62]. This intriguing dual role of the EPHs and ephrins in both promoting and suppressing cancer has been highlighted by various expression profiling tumor studies: for instance, EPHA4 overexpression promotes gliomas, breast and colorectal cancers whereas it suppresses lung cancer [26,38,50,59]. In addition, this dual role has been featured by the ability of the EPHs to switch from functioning as promoters to suppressors during cancer progression in the same tumor: EPHB expression is lost along the adenoma-carcinoma sequence, during colorectal carcinogenesis, due to the epigenetic silencing [15,64]. Therefore, the tissue type and tumor stage where a potential targeted therapy might be applied is crucial. To make things more complicated, members of the EPH family interact with one another; EPHB6, for instance, is capable of forming heterodimers with EPHA2, EPHB1, and EPHB4 [18,42,65].

The complexity that characterizes the mechanisms behind the EPH/ephrin system’s tumor-promoting and tumor-suppressing properties has been reported by our group in an extensive review of the literature. We emphasized the clinical significance of the various EPHs/ephrins expression’s alterations in solid tumors, pointing out that up- or downregulation of a certain member of the aforementioned biomolecules could possibly enhance tumorigenesis in a certain organ while suppressing it in another [66].

Indeed, targeting EPHA2 receptors might be a key strategy against breast cancer in the era of personalized medicine. EPHA2 overexpression is associated with hormone positive breast patients and is linked with adverse prognosis when present in this subgroup [67,68]. It is also followed by resistance to tamoxifen treatment, though its inhibition restores the sensitivity against this treatment in preclinical models [69]. Furthermore, EPHA2 overexpression synergizes with HER2 to promote breast cancer progression and correlates with worse OS and DFS in HER2 positive cancers; in addition, EPHA2 contributes to the development of resistance against the anti-HER2 monoclonal antibody trastuzumab, while its inhibition could restore sensitivity against this regimen [27,44]. Notably, our group and others [32] correlated EPHA2 overexpression with decreased OS and DFS in TNBC patients at a significant level. Last, as mentioned before, increased EPHA2 expression on endothelial cells promotes angiogenesis [17,28]. As a result of all of the above, targeted treatment against EPHA2 might be efficient in ER-positive and HER2-positive breast cancers as well as TNBCs. Likewise, targeted treatment against EPHA2 could be of value in combination with tamoxifen or trastuzumab in mutually EPHA2- and ER- or EPHA2- and HER2-positive subgroups of invasive breast cancer patients, respectively [17,26,44,69].

In conclusion, TNBCs are characterized by poor prognosis and lack of an available target therapy, leaving toxic chemotherapy as the only option [10,12,13,14]. Our immunohistochemical study identified a subgroup of TNBC patients that overexpressed EPHA2 and presented with an even higher risk of recurrence and worse overall prognosis, at a significant level. Therefore, EPHA2 might be effective as a biomarker for determining the most appropriate management, assessing prognosis, and designing a proper targeted treatment profitable for these patients. Our study also linked high EPHA4 expression with the presence of lymph node metastasis and high EPHA7 expression with a higher nuclear grade of tumor cells, both at no significant level though. All EPHs studied were upregulated in TNBC cell lines. More expression profiling studies in larger human cancer cohorts are needed to determine clinical significance in the EPH/ephrin field and then translate any new knowledge into effective personalized treatments.

## Figures and Tables

**Table 1:** Association of EPHA2 expression with selected clinicopathologic parameters in 52 triple-negative breast cancer (TNBC) patients (*p*-values calculated through the Fisher’s exact test).

Clinicopathologic Parameters	EPHA2 Expression		
	Low (%)	High (%)	*p*-Value
*N* = 52	26 (50.0)	26 (50.0)	
Age (mean ± SD; years)			0.1637
≤57.0 ± 12.6 years	9 (17.3)	15 (28.8)	
>57.0 ± 12.6 years	17 (32.7)	11 (21.2)	
Menopausal status			0.7645
Premenopausal	7 (13.7)	8 (15.7)	
Postmenopausal	19 (37.3)	17 (33.3)	
Histologic type			0.7712
Ductal	18 (34.6)	16 (30.8)	
Lobular	8 (15.4)	10 (19.2)	
Grade			0.7813
I + II	15 (28.9)	13 (25.0)	
III	11 (21.1)	13 (25.0)	
Nuclear grade			0.7813
I	15 (28.9)	13 (25.0)	
II + III	11 (21.1)	13 (25.0)	
Tumor size			1.0000
<2 cm	8 (15.4)	7 (13.5)	
>2 cm	18 (34.6)	19 (36.5)	
Lymph nodes			0.7793
Non-infiltrated	12 (23.1)	10 (19.2)	
Infiltrated	14 (26.9)	16 (30.8)	
Stage			0.6703
I	6 (11.5)	4 (7.7)	
II	16 (30.8)	16 (30.8)	
III	4 (7.7)	6 (11.5)	
Ki-67 protein status			0.0115
Below median value	17 (32.7)	7 (13.5)	
Over median value	9 (17.3)	19 (36.5)	

**Table 2:** Association of EPHA4 expression with selected clinicopathologic parameters in 52 triple-negative breast cancer (TNBC) patients (*p*-values calculated through the Fisher’s exact test).

Clinicopathologic Parameters	EPHA4 Expression		
	Low (%)	High (%)	*p*-Value
*N* = 52	27 (51.9)	25 (48.1)	
Age (mean ± SD; years)			0.7880
≤57.0 ± 12.6 years	13 (25.0)	11 (21.2)	
>57.0 ± 12.6 years	14 (26.9)	14 (26.9)	
Menopausal status			1.0000
Premenopausal	8 (15.4)	8 (15.4)	
Postmenopausal	19 (36.5)	17 (32.7)	
Histologic type			0.3917
Ductal	16 (30.8)	18 (34.6)	
Lobular	11 (21.2)	7 (13.5)	
Grade			0.5783
I + II	16 (30.8)	12 (23.1)	
III	11 (21.1)	13 (25.0)	
Nuclear grade			1.0000
I	15 (28.9)	13 (25.0)	
II + III	12 (23.1)	12 (23.1)	
Tumor size			0.5475
<2 cm	9 (17.3)	6 (11.5)	
>2 cm	18 (34.6)	19 (36.5)	
Lymph nodes			0.0546
Non-infiltrated	15 (28.8)	7 (13.5)	
Infiltrated	12 (23.1)	18 (34.6)	
Stage			0.8506
I	6 (11.5)	4 (7.7)	
II	16 (30.8)	16 (30.8)	
III	5 (9.6)	5 (9.6)	
Ki-67 protein status			0.7880
Below median value	13 (25.0)	11 (21.2)	
Over median value	14 (26.9)	14 (26.9)	

**Table 3:** Association of EPHA7 expression with selected clinicopathologic parameters in 52 triple-negative breast cancer (TNBC) patients (*p*-values calculated through the Fisher’s exact test).

Clinicopathologic Parameters	EPHA7 Expression		
	Low (%)	High (%)	*p*-Value
*N* = 52	20 (38.5)	32 (61.5)	
Age (mean ± SD; years)			1.0000
≤57.0 ± 12.6 years	9 (17.3)	15 (28.8)	
>57.0 ± 12.6 years	11 (21.2)	17 (32.7)	
Menopausal status			1.0000
Premenopausal	6 (11.5)	10 (19.2)	
Postmenopausal	14 (26.9)	22 (42.3)	
Histologic type			0.2439
Ductal	11 (21.2)	23 (44.2)	
Lobular	9 (17.3)	9 (17.3)	
Grade			1.0000
I + II	11 (21.2)	17 (32.7)	
III	9 (17.3)	15 (28.8)	
Nuclear grade			0.0889
I	14 (26.9)	14 (26.9)	
II + III	6 (11.5)	18 (34.6)	
Tumor size			0.3523
<2 cm	4 (7.7)	11 (21.2)	
>2 cm	16 (30.8)	21 (40.4)	
Lymph nodes			1.0000
Non-infiltrated	8 (15.4)	14 (26.9)	
Infiltrated	12 (23.1)	18 (34.6)	
Stage			0.6448
I	5 (9.6)	5 (9.6)	
II	12 (23.1)	20 (38.5)	
III	3 (5.8)	7 (13.5)	
Ki-67 protein status			0.3953
Below median value	11 (21.2)	13 (25.0)	
Over median value	9 (17.3)	19 (36.5)	

**Table 4:** Multivariate analysis for histologic type, grade, tumor size, lymph node status, Ki-67 status, and EPHA2 expression for OS.

Clinicopathologic Variables	Overall Survival	
	HR (95% CI)	*p*-Value
Histologic type (ductal/lobular)	0.774 (0.182–1.964)	0.7692
Grade (I + II/III)	0.576 (0.075–1.874)	0.4874
Tumor size (<2 cm/>2 cm)	1.202 (0.156–2.851)	0.8769
Lymph nodes (non-infiltrated/infiltrated)	0.557 (0.067–1.936)	0.4376
Ki-67 status (below/over median value)	10.921 (6.472–13.449)	0.0808
EPHA2 expression (low/high)	13.149 (7.352–18.777)	0.0041

**Table 5:** Multivariate analysis for histologic type, grade, tumor size, lymph node status, Ki-67 status, and EPHA2 expression for DFS.

Clinicopathologic Variables	Disease-Free Survival	
	HR (95% CI)	*p*-Value
Histologic type (ductal/lobular)	0.463 (0.127–1.082)	0.3377
Grade (I + II/III)	0.413 (0.132–1.076)	0.2159
Tumor size (<2 cm/>2 cm)	0.319 (0.094–0.956)	0.1619
Lymph nodes (non-infiltrated/infiltrated)	2.614 (1.123–5.995)	0.1859
Ki-67 status (below/over median value)	7.572 (4.661–13.559)	0.0481
EphA2 expression (low/high)	3.592 (1.767–5.892)	0.0232

## Data Availability

The data presented in this study are available upon request from the corresponding author.

## References

[1] Torre L.A., Bray F., Siegel R.L., Ferlay J., Lortet-Tieulent J., Jemal A. (2015). Global cancer statistics, 2012. CA Cancer J. Clin..

[2] Skibinski A., Kuperwasser C. (2015). The origin of breast tumor heterogeneity. Oncogene.

[3] Sotiriou C., Pusztai L. (2009). Gene-Expression Signatures in Breast Cancer. N. Engl. J. Med..

[4] Schnitt S.J. (2010). Classification and prognosis of invasive breast cancer: From morphology to molecular taxonomy. Mod. Pathol..

[5] Lips E.H., Mulder L., De Ronde J.J., Mandjes I.A.M., Koolen B.B., Wessels L.F.A., Rodenhuis S., Wesseling J. (2013). Breast cancer subtyping by immunohistochemistry and histological grade outperforms breast cancer intrinsic subtypes in predicting neoadjuvant chemotherapy response. Breast Cancer Res. Treat..

[6] Allison K.H. (2012). Molecular Pathology of Breast Cancer: What a pathologist needs to know. Am. J. Clin. Pathol..

[7] Kos Z., Dabbs D.J. (2015). Biomarker assessment and molecular testing for prognostication in breast cancer. Histopathology.

[8] Coates A.S., Winer E.P., Goldhirsch A., Gelber R.D., Gnant M., Piccart-Gebhart M., Thürlimann B., Senn H.-J. (2015). Panel Members. Tailoring therapies—Improving the management of early breast cancer: St Gallen International Expert Consensus on the Primary Therapy of Early Breast Cancer 2015. Ann. Oncol..

[9] Curigliano G., Burstein H.J., Winer E.P., Gnant M., Dubsky P., Loibl S., Colleoni M., Regan M.M., Piccart-Gebhart M., Senn H.-J. (2017). De-escalating and escalating treatments for early-stage breast cancer: The St. Gallen International Expert Consensus Conference on the Primary Therapy of Early Breast Cancer 2017. Ann. Oncol..

[10] Griffiths C.L., Olin J.L. (2012). Triple Negative Breast Cancer: A Brief Review of its Characteristics and Treatment Options. J. Pharm. Pract..

[11] Newman L.A., Reis-Filho J.S., Morrow M., Carey L.A., King T.A. (2014). The 2014 Society of Surgical Oncology Susan G. Komen for the Cure Symposium: Triple-Negative Breast Cancer. Ann. Surg. Oncol..

[12] Bose S. (2015). Triple-negative Breast Carcinoma: Morphologic and molecular subtypes. Adv. Anat. Pathol..

[13] Bianchini G., Balko J.M., Mayer I.A., Sanders M.E., Gianni L. (2016). Triple-negative breast cancer: Challenges and opportunities of a heterogeneous disease. Nat. Rev. Clin. Oncol..

[14] Lehmann B.D., Bauer J.A., Chen X., Sanders M.E., Chakravarthy A.B., Shyr Y., Pietenpol J.A. (2011). Identification of human triple-negative breast cancer subtypes and preclinical models for selection of targeted therapies. J. Clin. Investig..

[15] Pasquale E.B. (2010). Eph receptors and ephrins in cancer: Bidirectional signalling and beyond. Nat. Rev. Cancer.

[16] Barquilla A., Pasquale E.B. (2015). Eph Receptors and Ephrins: Therapeutic Opportunities. Annu. Rev. Pharmacol. Toxicol..

[17] Vaught D., Brantley-Sieders D.M., Chen J. (2008). Eph receptors in breast cancer: Roles in tumor promotion and tumor suppression. Breast Cancer Res..

[18] Boyd A.W., Bartlett P.F., Lackmann M. (2014). Therapeutic targeting of EPH receptors and their ligands. Nat. Rev. Drug Discov..

[19] Brantley-Sieders D.M. (2012). Clinical relevance of Ephs and ephrins in cancer: Lessons from breast, colorectal, and lung cancer profiling. Semin. Cell Dev. Biol..

[20] Chen J. (2012). Regulation of Tumor Initiation and Metastatic Progression by Eph Receptor Tyrosine Kinases. Advances in Cancer Research.

[21] Surawska H., Ma P.C., Salgia R. (2004). The role of ephrins and Eph receptors in cancer. Cytokine Growth Factor Rev..

[22] Xi H.-Q., Wu X.-S., Wei B., Chen L. (2012). Eph receptors and ephrins as targets for cancer therapy. J. Cell. Mol. Med..

[23] Zelinski D.P., Zantek N., Stewart J.C., Irizarry A.R., Kinch M. (2001). EphA2 overexpression causes tumorigenesis of mammary epithelial cells. Cancer Res..

[24] Ogawa K., Pasqualini R., A Lindberg R., Kain R., Freeman A.L., Pasquale E.B. (2000). The ephrin-A1 ligand and its receptor, EphA2, are expressed during tumor neovascularization. Oncogene.

[25] Fang W.B., Brantley-Sieders D.M., Parker M.A., Reith A.D., Chen J. (2005). A kinase-dependent role for EphA2 receptor in promoting tumor growth and metastasis. Oncogene.

[26] Brantley-Sieders D.M., Jiang A., Sarma K., Badu-Nkansah A., Walter D.L., Shyr Y., Chen J. (2011). Eph/Ephrin Profiling in Human Breast Cancer Reveals Significant Associations between Expression Level and Clinical Outcome. PLoS ONE.

[27] Brantley-Sieders D.M., Zhuang G., Hicks D., Bin Fang W., Hwang Y., Cates J.M., Coffman K., Jackson D., Bruckheimer E., Muraoka-Cook R.S. (2008). The receptor tyrosine kinase EphA2 promotes mammary adenocarcinoma tumorigenesis and metastatic progression in mice by amplifying ErbB2 signaling. J. Clin. Investig..

[28] Kaenel P., Mosimann M., Andres A.-C. (2012). The multifaceted roles of Eph/ephrin signaling in breast cancer. Cell Adhes. Migr..

[29] Husa A.-M., Magić Ž., Larsson M., Fornander T., Pérez-Tenorio G. (2016). EPH/ephrin profile and EPHB2 expression predicts patient survival in breast cancer. Oncotarget.

[30] Nikas I., Ryu H.S., Theocharis S. (2018). Viewing the Eph receptors with a focus on breast cancer heterogeneity. Cancer Lett..

[31] Toosi B.M., El Zawily A., Truitt L., Shannon M., Allonby O., Babu M., DeCoteau J., Mousseau D., Ali M., Freywald T. (2018). EPHB6 augments both development and drug sensitivity of triple-negative breast cancer tumours. Oncogene.

[32] Song W., Hwang Y., Youngblood V.M., Cook R.S., Balko J.M., Chen J., Brantley-Sieders D.M. (2017). Targeting EphA2 impairs cell cycle progression and growth of basal-like/triple-negative breast cancers. Oncogene.

[33] Hachim I.Y., Villatoro M., Canaff L., Hachim M.Y., Boudreault J., Haiub H., Ali S., Lebrun J.-J. (2017). Transforming Growth Factor-beta Regulation of Ephrin Type-A Receptor 4 Signaling in Breast Cancer Cellular Migration. Sci. Rep..

[34] Mylona E., Vamvakaris I., Giannopoulou I., Theohari I., Papadimitriou C., Keramopoulos A., Nakopoulou L. (2013). An immunohistochemical evaluation of the proteins Wnt1 and glycogen synthase kinase (GSK)-3β in invasive breast carcinomas. Histopathology.

[35] Elston C., Ellis I. (1991). pathological prognostic factors in breast cancer. I. The value of histological grade in breast cancer: Experience from a large study with long-term follow-up. Histopathology.

[36] Rakha E.A., El-Sayed M.E., Lee A.H.S., Elston C.W., Grainge M.J., Hodi Z., Blamey R.W., Ellis I.O. (2008). Prognostic Significance of Nottingham Histologic Grade in Invasive Breast Carcinoma. J. Clin. Oncol..

[37] Giuliano A.E., Connolly J.L., Edge S.B., Mittendorf E.A., Rugo H.S., Solin L.J., Weaver D.L., Winchester D.J., Hortobagyi G.N. (2017). Breast Cancer-Major changes in the American Joint Committee on Cancer eighth edition cancer staging manual. CA Cancer J. Clin..

[38] Giaginis C., Tsoukalas N., Bournakis E., Alexandrou P., Kavantzas N., Patsouris E., Theocharis S. (2014). Ephrin (Eph) receptor A1, A4, A5 and A7 expression in human non-small cell lung carcinoma: Associations with clinicopathological parameters, tumor proliferative capacity and patients’ survival. BMC Clin. Pathol..

[39] Giaginis C., Tsourouflis G., Zizi-Serbetzoglou A., Kouraklis G., Chatzopoulou E., Dimakopoulou K., Theocharis S.E. (2009). Clinical Significance of Ephrin (Eph)-A1, -A2, -A4, -A5 and -A7 Receptors in Pancreatic Ductal Adenocarcinoma. Pathol. Oncol. Res..

[40] Karidis N.P., Giaginis C., Tsourouflis G., Alexandrou P., Delladetsima I., Theocharis S. (2011). Eph-A2 and Eph-A4 expression in human benign and malignant thyroid lesions: An immunohistochemical study. Med Sci. Monit..

[41] Theocharis S., Klijanienko J., Giaginis C., Alexandrou P., Patsouris E., Sastre-Garau X. (2013). Ephrin Receptor (Eph)-A1, -A2, -A4 and -A7 Expression in Mobile Tongue Squamous Cell Carcinoma: Associations with Clinicopathological Parameters and Patients Survival. Pathol. Oncol. Res..

[42] Fox B.P., Kandpal R.P. (2009). EphB6 receptor significantly alters invasiveness and other phenotypic characteristics of human breast carcinoma cells. Oncogene.

[43] Truitt L., Freywald T., DeCoteau J., Sharfe N., Freywald A. (2010). The EphB6 Receptor Cooperates with c-Cbl to Regulate the Behavior of Breast Cancer Cells. Cancer Res..

[44] Zhuang G., Brantley-Sieders D.M., Vaught D., Yu J., Xie L., Wells S., Jackson D., Muraoka-Cook R., Arteaga C., Chen J. (2010). Elevation of Receptor Tyrosine Kinase EphA2 Mediates Resistance to Trastuzumab Therapy. Cancer Res..

[45] Edwards D.N., Ngwa V.M., Wang S., Shiuan E., Brantley-Sieders D.M., Kim L.C., Reynolds A.B., Chen J. (2017). The receptor tyrosine kinase EphA2 promotes glutamine metabolism in tumors by activating the transcriptional coactivators YAP and TAZ. Sci. Signal..

[46] Youngblood V.M., Kim L.C., Edwards D., Hwang Y., Santapuram P.R., Stirdivant S.M., Lu P., Ye F., Brantley-Sieders D.M., Chen J. (2016). The Ephrin-A1/EPHA2 Signaling Axis Regulates Glutamine Metabolism in HER2-Positive Breast Cancer. Cancer Res..

[47] Fox B.P., Kandpal R.P. (2004). Invasiveness of breast carcinoma cells and transcript profile: Eph receptors and ephrin ligands as molecular markers of potential diagnostic and prognostic application. Biochem. Biophys. Res. Commun..

[48] Wu D., Suo Z., Kristensen G.B., Li S., Troen G., Holm R., Nesland J.M. (2004). Prognostic value of EphA2 and EphrinA-1 in squamous cell cervical carcinoma. Gynecol. Oncol..

[49] Thaker P.H., Deavers M., Celestino J., Thornton A., Fletcher M.S., Landen C.N., Kinch M., Kiener P.A., Sood A.K. (2004). EphA2 Expression Is Associated with Aggressive Features in Ovarian Carcinoma. Clin. Cancer Res..

[50] Herrem C.J., Tatsumi T., Olson K.S., Shirai K., Finke J.H., Bukowski R.M., Zhou M., Richmond A.L., Derweesh I., Kinch M. (2005). Expression of EphA2 is prognostic of disease-free interval and overall survival in surgically treated patients with renal cell carcinoma. Clin. Cancer Res..

[51] Holm R., Knopp S., Suo Z., Trope C., Nesland J.M. (2007). Expression of EphA2 and EphrinA-1 in vulvar carcinomas and its relation to prognosis. J. Clin. Pathol..

[52] Wang L.-F., Fokas E., Bieker M., Rose F., Rexin P., Zhu Y., Pagenstecher A., Engenhart-Cabillic R., An H.-X. (2008). Increased expression of EphA2 correlates with adverse outcome in primary and recurrent glioblastoma multiforme patients. Oncol. Rep..

[53] Shao Z., Zhang W.-F., Chen X., Shang Z.-J. (2008). Expression of EphA2 and VEGF in squamous cell carcinoma of the tongue: Correlation with the angiogenesis and clinical outcome. Oral Oncol..

[54] Kamat A.A., Coffey D., Merritt W.M., Nugent E., Urbauer D., Lin Y.G., Edwards C., Broaddus R., Coleman R.L., Sood A.K. (2009). EphA2 overexpression is associated with lack of hormone receptor expression and poor outcome in endometrial cancer. Cancer.

[55] Yuan W., Chen Z., Wu S., Ge J., Chang S., Wang X., Chen J., Chen Z. (2009). Expression of EphA2 and E-cadherin in Gastric Cancer: Correlated with Tumor Progression and Lymphogenous Metastasis. Pathol. Oncol. Res..

[56] Miyazaki T., Kato H., Fukuchi M., Nakajima M., Kuwano H. (2003). EphA2 overexpression correlates with poor prognosis in esophageal squamous cell carcinoma. Int. J. Cancer.

[57] Predictive Value of the EphA2 Receptor Tyrosine Kinase in Lung Cancer Recurrence and Survival|Clinical Cancer Research. https://clincancerres.aacrjournals.org/content/9/2/613.long.

[58] Saito T., Masuda N., Miyazaki T., Kanoh K., Suzuki H., Shimura T., Asao T., Kuwano H. (2004). Expression of EphA2 and E-cadherin in colorectal cancer: Correlation with cancer metastasis. Oncol. Rep..

[59] Nakada M., Hayashi Y., Hamada J.-I. (2011). Role of Eph/ephrin tyrosine kinase in malignant glioma. Neuro-Oncology.

[60] Oki M., Yamamoto H., Taniguchi H., Adachi Y., Imai K., Shinomura Y. (2008). Overexpression of the receptor tyrosine kinase EphA4 in human gastric cancers. World J. Gastroenterol..

[61] Saintigny P., Peng S., Zhang L., Sen B., Wistuba I.I., Lippman S.M., Girard L., Minna J.D., Heymach J.V., Johnson F.M. (2012). Global Evaluation of Eph Receptors and Ephrins in Lung Adenocarcinomas Identifies EphA4 as an Inhibitor of Cell Migration and Invasion. Mol. Cancer Ther..

[62] Lisabeth E.M., Falivelli G., Pasquale E.B. (2013). Eph Receptor Signaling and Ephrins. Cold Spring Harb. Perspect. Biol..

[63] Noblitt L.W., Bangari D.S., Shukla S., Knapp D.W., I Mohammed S., Kinch M.S., Mittal S.K. (2004). Decreased tumorigenic potential of EphA2-overexpressing breast cancer cells following treatment with adenoviral vectors that express EphrinA1. Cancer Gene Ther..

[64] Batlle E., Bacani J., Begthel H., Jonkheer S., Gregorieff A., Van De Born M., Malats N., Sancho E., Boon E., Pawson T. (2005). EphB receptor activity suppresses colorectal cancer progression. Nature.

[65] Truitt L., Freywald A. (2011). Dancing with the dead: Eph receptors and their kinase-null partnersThis paper is one of a selection of papers published in a Special Issue entitled CSBMCB 53rd Annual Meeting—Membrane Proteins in Health and Disease, and has undergone the Journal’s usual peer review process. Biochem. Cell Biol..

[66] Pergaris A., Danas E., Goutas D., Sykaras A., Soranidis A., Theocharis S. (2021). The Clinical Impact of the EPH/Ephrin System in Cancer: Unwinding the Thread. Int. J. Mol. Sci..

[67] Martin K.J., Patrick D.R., Bissell M.J., Fournier M.V. (2008). Prognostic Breast Cancer Signature Identified from 3D Culture Model Accurately Predicts Clinical Outcome across Independent Datasets. PLoS ONE.

[68] Pan M. (2005). Overexpression of EphA2 gene in invasive human breast cancer and its association with hormone receptor status. J. Clin. Oncol..

[69] Gokmen-Polar Y., Toroni R.A., Hocevar B.A., Badve S., Zhao Q., Shen C., Bruckheimer E.M., Kinch M., Miller K.D. (2010). Dual targeting of EphA2 and ER restores tamoxifen sensitivity in ER/EphA2-positive breast cancer. Breast Cancer Res. Treat..

